# Sungai Lembing’s green tourism: Pioneering the future of resource-based urban renewal

**DOI:** 10.1016/j.xinn.2024.100578

**Published:** 2024-01-12

**Authors:** Haoxuan Yu, Ginura Gunaratna, Izni Zahidi, Chow Ming Fai

**Affiliations:** 1 Department of Civil Engineering, School of Engineering, Monash University Malaysia, Bandar Sunway, Selangor 47500, Malaysia

## How did Sungai Lembing pave its path to a green evolution?

Sungai Lembing, a resource-based town situated in Pahang, Malaysia, has historically positioned itself as a pivotal hub in the global tin mining sector. Geologically, Sungai Lembing’s history dates back to the Paleozoic and Mesozoic eras. These eras were marked by the closure of the Tethyan Ocean and the convergence of continental landmasses from the eastern part of the Gondwana continent. This complex geological background resulted in a wide variety of mineral resources. The collision of tectonic plates is believed to have led to the creation of Sungai Lembing’s tin-rich soil.^1^ The discovery of the world’s most extensive underground tin mines in the late 19th century not only propelled the town into the global spotlight but also catalyzed a dramatic economic transformation.^2^ The influx of investors and miners led to a proliferation of new economic activities in the region, ranging from trading to the development of infrastructure. The rapid emergence of infrastructure, including transport and processing facilities, not only improved tin extraction and export but also reshaped the town’s landscape and daily life.

However, mirroring the volatile nature of commodities, Sungai Lembing’s prosperity was not destined to last. Due to global market shifts and a decrease in demand, the mid-1980s witnessed a sharp plummet in tin prices. By 1986, this downturn forced the once-thriving mines of Sungai Lembing to halt operations, casting a shadow of financial uncertainty over the town.^3^ In addition to facing financial instability, the Sungai Lembing tin mine had long employed mining techniques akin to the stope caving method, relying on traditional track mining equipment such as manually operated drills for their operations (https://www.nst.com.my/news/nation/2019/05/485428/good-old-days-sg-lembing). These practices led to significant environmental degradation and geological hazards, severely damaging the local vegetation and disrupting the ecosystem. The loss of vegetation, a cornerstone of the local ecosystem, led to increased soil erosion and a heightened risk of landslides. Furthermore, the environmental degradation diminished the natural beauty of the area and adversely affected the well-being and quality of life for the community. Addressing these environmental and safety challenges is essential for restoring the region’s ecological balance and ensuring a sustainable future for Sungai Lembing’s community.

Sungai Lembing has demonstrated remarkable resilience, confronting the twin challenges of economic recessions and environmental degradation with unyielding fortitude. As the 20th century drew to a close, the town’s leadership and community agencies initiated efforts to mitigate the environmental damage resulting from decades of mining activities.^4^ This strategic pivot not only focused on mitigating the environmental impact but also steered the economy toward a burgeoning green tourism sector, leveraging the town’s natural landscapes and cultural heritage (https://en.wikipedia.org/wiki/sungai_lembing).

**Figure 1:**
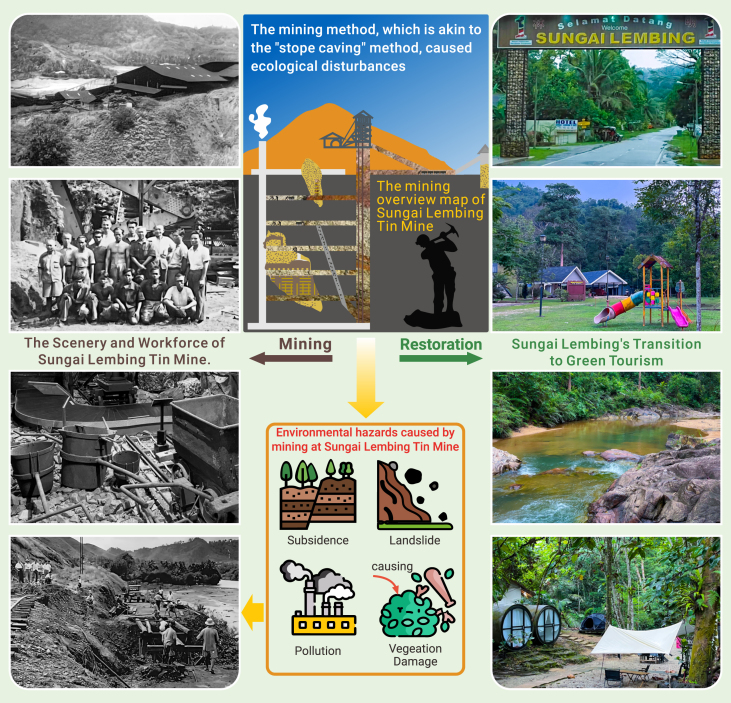
From extraction to green tourism: The transformation of Sungai Lembing

Sungai Lembing’s dedication to ecological restoration took shape through a comprehensive reforestation initiative, targeting more than just cosmetic changes. This effort aimed to restore indigenous vegetation, stabilize erosion-prone soil, and encourage the return of wildlife, reflecting a deep commitment to healing the landscape. Alongside ecological restoration, Sungai Lembing also sought economic revival by embracing its mining heritage. The town transformed historical mining sites into cultural exhibits, thereby integrating its rich history into a new economic framework. This reimagined identity invites travelers to form a deep bond with the land, allowing them to immerse themselves in its rich history and cultural legacy (please see Figure 1).

## What can resource-based communities learn from Sungai Lembing's evolution?

Sungai Lembing has remarkably transitioned from a mining-centric locale to an exemplary model of rejuvenation, emphasizing environmental rehabilitation and a shift toward green tourism. Unlike many resource-based locales with their own success narratives, Sungai Lembing’s approach is distinguished by its strategic focus on preserving and leveraging its natural resources for sustainable development. Sungai Lembing stands as a testament to the possibility of harmonizing economic development with ecological stewardship, offering valuable lessons for similar communities globally.

### Charting a defined path ahead

Sungai Lembing demonstrated clarity in charting its transformative journey, with strong government support and community resolve. In 1988, just a year after the Pahang state government ceased operations at the Sungai Lembing tin mine, an ambitious pivot toward ecotourism was proposed. Capitalizing on its rich heritage, Sungai Lembing has skillfully preserved numerous cultural landmarks from the early 20th century, turning its mining sites into a narrative of the town’s tin mining saga (https://www.penang-insider.com/sungai-lembing-things-to-do/#google_vignette). These heritage sites, along with the town’s picturesque landscapes and pristine natural attractions such as the Rainbow Waterfall, captivate a diverse audience, ranging from enthusiasts of Malaysia’s mining history to international nature lovers. Drawing from Sungai Lembing’s journey, other resource-based locales can harness their unique attributes to develop a well-defined vision and strategic plan for their future.

### Economic and environmental harmony

Sungai Lembing illustrates the balance between human endeavors and environmental conservation in green tourism. In charting its new economic trajectory, the town meticulously incorporated ecological restoration initiatives, such as implementing measures to mitigate the effects of residual mining waste.^5^ This balanced approach not only rejuvenated the town’s natural landscapes but also provided a simultaneous boost to the local economy. The town prioritized environmental considerations rather than sidelining them in pursuit of economic growth. They recognized that a thriving environment can, in turn, foster economic vitality. This cyclical strategy, where environmental conservation fuels economic gains, and vice versa, demonstrates a sustainable growth model worthy of emulation. It calls upon these regions to proactively recalibrate their development strategies, focusing on the lasting benefits of sustainable growth over transient economic advantages. At its core, communities that equally value economic and environmental targets set themselves on a path toward a future that is both prosperous and sustainable.

### Dedication and tenacity

The evolution of Sungai Lembing is a narrative of steadfast commitment. For over three decades since the mine’s closure in 1986, the town has unwaveringly pursued a vision to transition from its mining legacy to a hub of green tourism. This journey of transformation has been marked by a clear strategy and an unflinching resolve to eschew quick fixes in favor of long-term sustainability. In the process, Sungai Lembing has become a paragon for environmental conservation and economic revitalization. Its perseverance in nurturing a tourism industry that celebrates its unique mining heritage has not only reinvigorated the local economy but has also redefined its identity. Sungai Lembing’s story is one of resilience, highlighting the benefits of steadfast commitment, a clear vision, and the patience to realize it. As such, Sungai Lembing serves as an inspiring example for communities globally, demonstrating the fruitful outcomes achievable through unwavering commitment to a greener and more sustainable future.

## How can resource-based communities navigate toward a sustainable future?

Sungai Lembing’s transformation from a mining-centric economy to a model of green tourism offers a valuable roadmap for resource-based localities aiming for a sustainable future. The foundation of this study lies in three key pillars: strategic planning, active community involvement, and committed environmental stewardship, each playing a pivotal role in driving successful economic transitions. This journey, spanning three decades, showcases the power of unwavering commitment to ecological conservation and the innovative repurposing of cultural heritage. For communities facing similar challenges, the lesson is unequivocal: a long-term vision focused on environmental and economic sustainability trumps the allure of short-term economic boons.

In conclusion, the Sungai Lembing case study serves as a harbinger of hope and a strategic guide for like-minded communities worldwide with similar challenges. It affirms that by combining visionary leadership, active community engagement, and persistent dedication, it is feasible to strike a harmonious balance between robust economic development and environmental integrity. This case underscores the practicality of achieving a harmonious balance between robust economic development and the preservation of environmental integrity, offering a model for sustainable progress.
